# Gene Expression Profile Analysis of Human Epidermal Keratinocytes Expressing Human Papillomavirus Type 8 E7

**DOI:** 10.3389/pore.2022.1610176

**Published:** 2022-05-18

**Authors:** Xianzhen Chen, Ma Li, Yi Tang, Qichang Liang, Chunting Hua, Huiqin He, Yinjing Song, Hao Cheng

**Affiliations:** ^1^ Department of Dermatology and Venereology, Zhejiang University School of Medicine Sir Run Run Shaw Hospital, Hangzhou, China; ^2^ Department of Dermatology, Zhejiang Provincial People’s Hospital, People’s Hospital of Hangzhou Medical College, Hangzhou, China; ^3^ Department of Gastroenterology, Zhejiang University School of Medicine Sir Run Run Shaw Hospital, Hangzhou, China

**Keywords:** RNA-sequencing, HPV 8E7, immune regulations, metabolic reprogramming, epigenetic modifications

## Abstract

**Background:** Human papillomavirus type 8 (HPV8) has been implicated in the progress of non-melanoma skin cancers and their precursor lesions. The HPV8 E7 oncoprotein plays a key role in the tumorigenesis of HPV-associated cutaneous tumors. However, the exact role of HPV8 E7 in human epidermal carcinogenesis has not been fully elucidated.

**Methods:** To investigate the potential carcinogenic effects of HPV8 E7 on epithelial cells, we used RNA-sequencing technology to analyze the gene expression profile of HPV8 E7-overexpressed normal human epidermal keratinocytes (NHEKs).

**Results:** RNA-sequencing revealed 831 differentially expressed genes (DEGs) between HPV8 E7-expressing NHEKs and control cells, among which, 631 genes were significantly upregulated, and 200 were downregulated. Gene ontology annotation enrichment analysis showed that HPV8 E7 mainly affected the expression of genes associated with protein heterodimerization activity, DNA binding, nucleosomes, and nucleosome assembly. Kyoto Encyclopedia of Genes and Genomes pathway enrichment analysis revealed that overexpression of HPV8 E7 affected the expression of gene clusters associated with viral carcinogenesis and transcriptional misregulation in cancer and necroptosis signaling pathways that reportedly play crucial roles in HPV infection promotion and cancer progression. We also found the DEGs, such as *HKDC1* and *TNFAIP3*, were associated with epigenetic modifications, immune regulation, and metabolic pathways.

**Conclusion:** Our results demonstrate that the pro-carcinogenic effect of HPV8 expression in epithelial cells may be attributed to the regulatory effect of oncogene E7 on gene expression associated with epigenetic modifications and immune and metabolic status-associated gene expression. Although our data are based on an *in vitro* experiment, it provides the theoretical evidence that the development of squamous cell carcinoma can be caused by HPV.

## Introduction

Skin cancer incidence has rapidly increased over the years and is generally divided into malignant melanoma and non-melanoma skin cancer (NMSC), the latter being subdivided into cutaneous squamous cell carcinoma (SCC) and basal cell carcinoma [1]. To date, more than 200 human papillomavirus (HPV) genotypes have been discovered; these viruses can infect the skin and mucosa of several animal species [2]. HPVs are ubiquitous DNA viruses, and HPV infection, especially with high-risk subtypes, can mediate skin tumorigenesis [3]. HPV8, belonging to beta-genus HPV, is suspected to be carcinogenic in some skin cancer types, but the underlying pathological mechanisms of these cancers remain poorly understood [4].

HPV8 E6 and E7 reportedly promote the autophagic degradation of cellular checkpoint kinase-1, which may contribute to the oncogenic potential of the virus [5]. Furthermore, HPV8 E7 expression in the murine epidermis under the control of keratin-14 promoter showed that the E7 protein acts carcinogenically in mice by inducing the invasion of basal keratinocytes, which are regulated by the extracellular protein fibronectin [6]. However, the potential molecular mechanisms and the downstream transcriptional modulation effects of HPV8 E7-infected keratinocytes remain unclear [7, 8].

Changes in the epigenetic modifications, immune response, and metabolic status are associated with tumor occurrence due to HPV infection [9]. For example, clear CpG island methylation occurs in HPV-induced cutaneous warts [10]. Furthermore, recent studies have suggested that the HPV oncogenes E5, E6, and E7 play critical roles in the development of chronic inflammation through various mechanisms and can affect the redox homeostasis of host cells, inducing oxidative stress, which may promote viral integration and cancer development [11, 12]. Moreover, the oncoproteins HPV16 E6 and E7 regulate the cell metabolism to satisfy the energy demands necessary for persistent infection and development of cervical cancer [13]. However, the gene expression profiles of the oncogene HPV8 E7 in human epidermal keratinocytes remain unclear.

Currently, RNA-sequencing technology is widely used to detect transcriptome profiling in cancer research and therapy such as biomarker discovery and cancer heterogeneity, drug resistance, cancer microenvironment, and immunotherapy [14]. However, the transcriptome signatures in HPV8 E7-infected epithelial cells remain unclear. In our study, we took advantage of RNA-sequencing technology and the enrichment analysis of gene ontology (GO) and Kyoto Encyclopedia of Genes and Genomes (KEGG) and discovered numerous significantly altered gene transcriptional landscapes that could be clustered into epigenetic modifications, immune regulations, and metabolic reprogramming. This study will elucidate the understanding of the tumorigenic mechanisms of HPV8 E7 expression.

## Materials and Methods

### Cells and Reagents

Normal human epidermal keratinocyte (NHEK) cells were purchased from the American Type Culture Collection (ATCC, No. PCS-200-010). EpiLife Media (60 µM calcium) supplemented with the HKGS kit (S001K, Thermo Fisher) was used for cell culture, and cells were cultured at 37°C in a 5% CO_2_ atmosphere. Complementary DNA (cDNA) for HPV8 E7 was amplified from the plasmid pCDNA3.1-HPV-8E7, which was a gift from Prof. Angel Alonso (German Cancer Research Centre, Heidelberg, Germany) and inserted into the P23 pHAGE-fEF1a-IRES-ZsGreen lentiviral vector with a C-terminal FLAG tag for lentiviral packaging.

### Construction of Cell Line stably Overexpressing HPV8 E7

The HPV8 E7 overexpression cell line was constructed as described previously with a minor modification [15]. In brief, the HPV8 E7 lentivirus-overexpressing plasmid and packaging plasmids were co-transfected into HEK293T cells using the Lipofectamine 3000 transfection reagent for packaging. The supernatant of the cells was collected, combined, and centrifuged at 2,000 rpm for 5 min and then filtered using a filter with a diameter of 45 μm. The filtrate was determined by titer and stored at −80 °C until use. For lentiviral infection, 1 × 105 NHEK cells per well (a total of three independent wells) were seeded onto a 12-well plate overnight, and 1 ml of the lentiviral fluid was added to the cells for 4 h and 100 μg/ml of polybrene was added to enhance infection efficiency. For the control group, 1 × 105 NHEK cells per well (a total of three independent wells) were added with 1 ml of P23 pHAGE-fEF1a-IRES-ZsGreen control lentivirus. After 4 h, the virus medium was removed, and 1 ml of fresh complete medium was added. The infection efficiency was detected by flow cytometry after 48 h. The construct was confirmed *via* DNA sequencing.

### RNA Extraction

TRIzol reagent (Invitrogen, Life Technologies, United States) was used to isolate the total RNAs from the NHEK cells infected with the HPV8 E7-overexpression lentivirus or the control virus, and the quality and concentration of the purified total RNAs were evaluated using a Nanodrop spectrophotometer (Thermo Scientific Technology, United States).

### Library Construction and Sequencing

cDNA library construction and transcriptome sequencing were conducted by Lianchuan Biotechnology Co., Ltd. (Hangzhou, China) as previously described [16]. The Agilent Bioanalyzer 2100 (Santa Clara, CA, United States) was used to detect the RNA integrity. According to the standard procedure of Illumina (protocol # 15008136), 1 μg of the total RNA from each sample was used for each sequencing library. To minimize the potential ribosome RNA (rRNA) contamination, rRNAs were removed *via* a selection procedure using Poly (A) containing mRNA. The Experion DNA 1K chip was used to evaluate the libraries’ quality. After standard procedures including cDNA generation, end repair, A-tailing, adaptor ligation, and PCR amplification, the cDNAs were sequenced on an Illumina NovaSeq 6000 platform.

### Functional and Pathway Enrichment Analysis of Differentially Expressed Genes

The Database for Annotation, Visualization, and Integrated Discovery (DAVID) database was used for KEGG pathway and GO analyses of DEGs, as described previously [15, 17]. In brief, a false discovery rate with a threshold ≤0.05 was used to determine the *p*-value in multiple tests and ClusterProfiler R (3.4.4) software was used to visualize the KEGG pathway enrichment (http://www.genome.jp/kegg/) and GO terms (http://www.geneontology.org/).

### Statistical Analysis

All statistical analyses were performed with GraphPad Prism 8 (GraphPad Software Inc., La Jolla, CA, United States). Data were expressed as mean ± standard error of mean (S.E.M) and are representative of three independent experiments. The threshold criteria of DEGs were set as log2 fold-change value >1 and *p*-value < 0.05, and log (fold change) value >2. Significant differences were calculated by using an unpaired two-tailed Student’s *t* test, and *p* value < 0.05 was considered statistically significant.

## Results

### Overexpression of HPV8 E7 Affects the Gene Expression Profile in NHEKs

**FIGURE 1:**
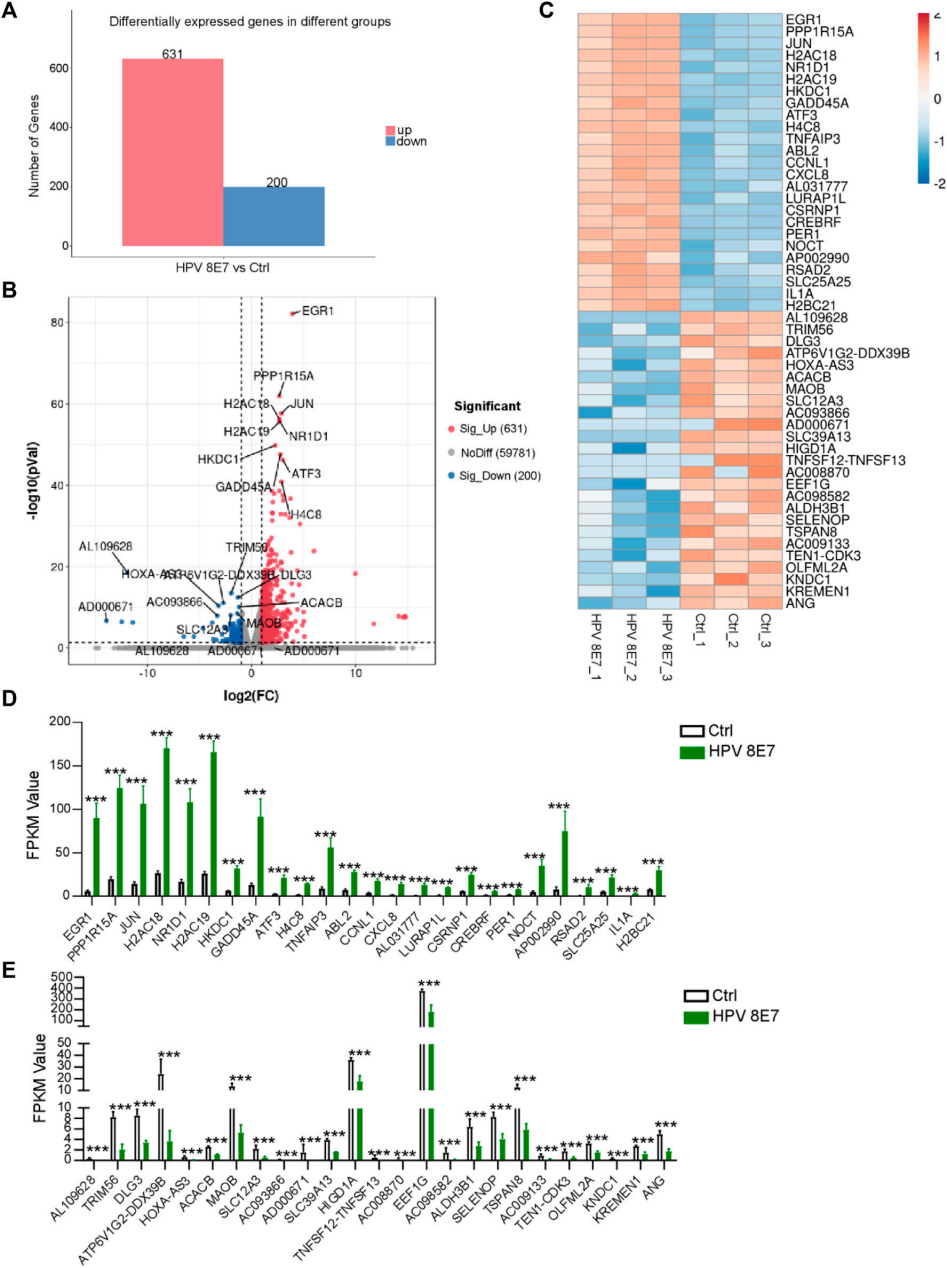
Overexpression of HPV8 E7 affected the gene expression profile in NHEKs. HPV8 E7 was overexpressed in NHEKs using the lentivirus system and the gene expression profiles were detected by the RNA-seq method. **(A)** Histogram representation of DEGs upregulated or downregulated in the control and HPV8 E7-overexpressed NHEK cells. **(B,C)** Volcano plot **(B)** and heatmap **(C)** representation of the 25 most significantly upregulated or downregulated genes in HPV8 E7-overexpressed NHEK cells compared with those in the controls. **(D)** Fragments per kilobase million (FPKM) values of the 25 most significantly upregulated genes in HPV8 E7-overexpressed NHEK cells compared with controls. **(E)** FPKM values of the 25 most significantly downregulated genes in HPV8 E7-overexpressed NHEK cells compared with those in the controls.

To investigate the potential carcinogenic effects of HPV8 E7 expression, we took advantage of RNA-sequencing technology to detect the gene expression profile of NHEK cells after HPV8 E7 overexpression and discovered 831 DEGs in the HPV8 E7-overexpressing NHEK cells compared with those in the controls (Figure 1A). Among the DEGs, 631 genes were significantly upregulated, and 200 were downregulated (Figure 1A). Furthermore, genes such as *EGR1*, *PPP1R15A*, *JUN,* and *H2AC18* were found to be the most significantly upregulated genes and genes including AL109628, TRIM56, *DLG3*, and *ATP6V1G2-DDX39B* were the most significantly downregulated upon HPV8 E7 overexpression (Figures 1B,C). Fragments per kilobase of exon model per million mapped fragments (FPKM) values are shown in Figure 1D,E.

### GO Enrichment Analysis of DEGs Affected by HPV8 E7 Overexpression

**FIGURE 2:**
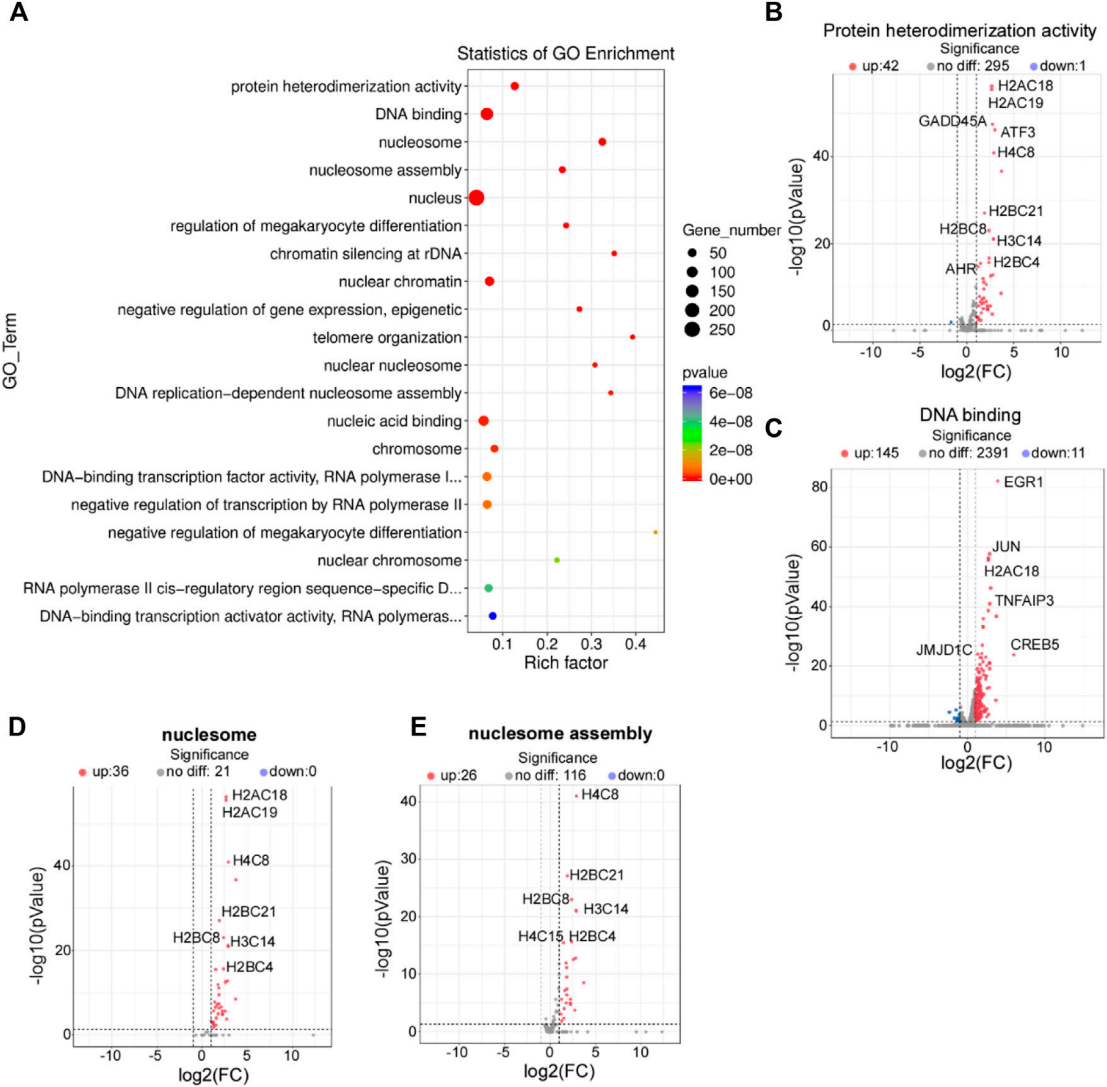
GO enrichment analysis of DEGs affected by HPV8 E7 overexpression. **(A)** Scatter plot for GO analysis. **(B)** Volcano plot representation of DEGs related to protein heterodimerization activity. **(C)** Volcano plot representation of DEGs related to DNA binding. **(D)** Volcano plot representation of DEGs related to nucleosomes. **(E)** Volcano plot representation of DEGs related to nucleosome assembly. See also Supplementary Figure S1.

To determine the biological function of HPV8 E7 in NHEKs, GO analysis was performed to functionally annotate and classify the DEGs. According to the enrichment factor classification, protein heterodimerization activity, DNA binding, nucleosomes, and nucleosome assembly were the most significantly enriched (Figure 2A). All 44 DEGs, including *A2AC18*, *H2AC19*, *GADD45*, *ATF3*, and *H4C8*, associated with protein heterodimerization activity were dramatically increased after HPV8 E7 expression (Supplementary Figure S1A; Figure 2B). Furthermore, a total of 50 DEGs associated with DNA binding were detected, 39 among which were significantly upregulated, including *EGR1*, *JUN*, *H2AC18*, *TNFAIP3*, and *CREB5*; 11 genes were downregulated, including *ZNF664*, *MYCN*, *PITX3*, *APOC0944,* and *WBP2NL* in HPV8 E7-infected NHEKs compared with those in the controls (Supplementary Figure S1B; Figure 2C). HPV8 E7 inhibited 36 genes, including *H2AC18*, *H2AC19*, *H4C8*, *H2BC21,* and *H2BC8*, which could be enriched into nucleosomes (Supplementary Figure S1C; Figure 2D). Furthermore, 26 genes, including *H4C8*, *H2BC21*, *H2BG8*, *H3C14,* and *H2BC4*, associated with nucleosome assembly were markedly downregulated upon HPV8 E7 expression (Supplementary Figure S1D; Figure 2E).

### KEGG Pathway Enrichment Analysis of DEGs Affected by HPV8 E7 Overexpression

**FIGURE 3:**
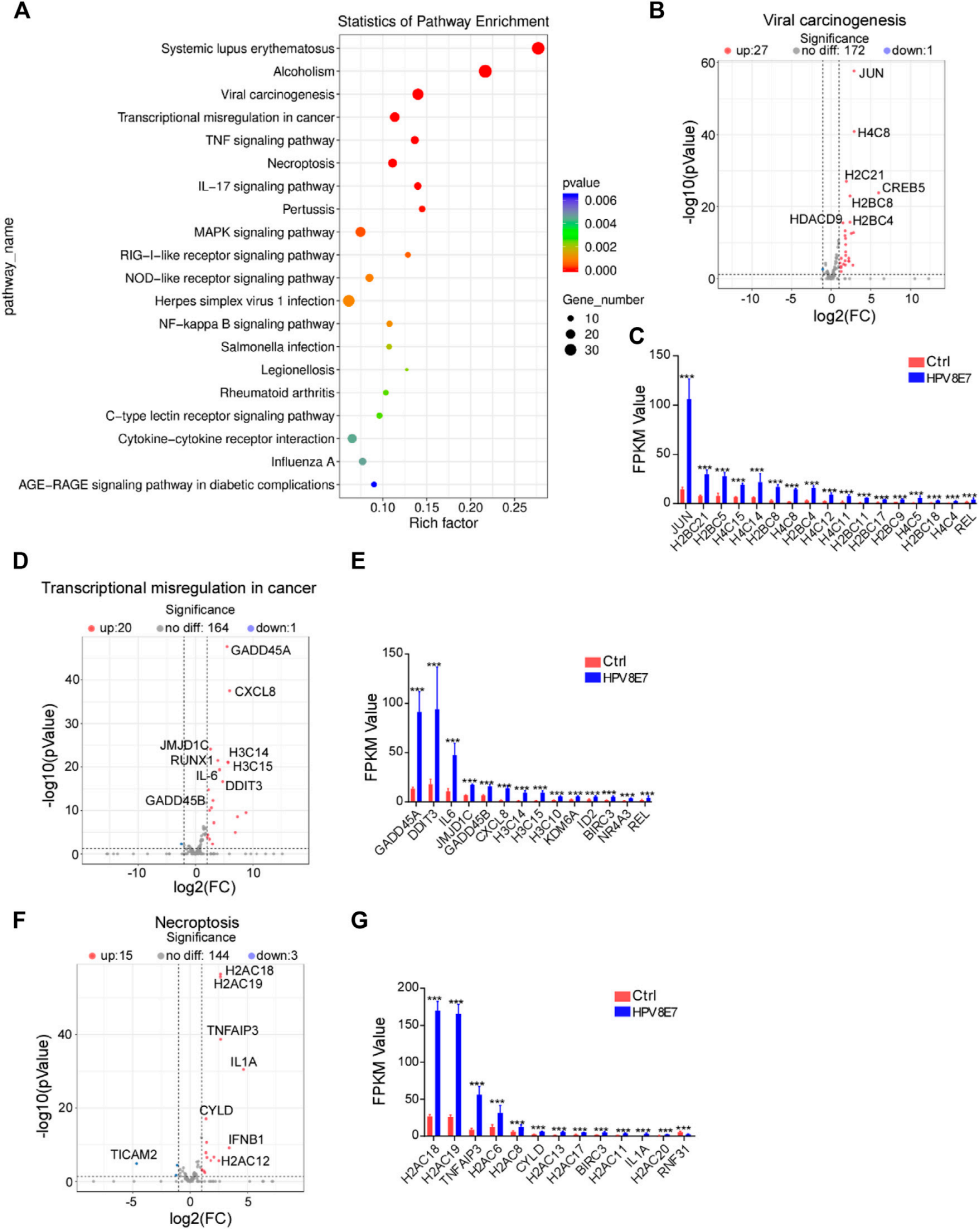
KEGG pathway enrichment analysis of DEGs affected by HPV8 E7 overexpression. **(A)** Scatter plot showing the enrichment analysis of the KEGG pathway. **(B,C)** Volcano plot **(B)** and FPKM value >1 **(B)** representation of DEGs related to viral carcinogenesis. **(D,E)** Volcano plot **(D)** and FPKM value >1 **(E)** representation of DEGs related to transcriptional misregulation in cancer. **(F,G)** Volcano plot **(F)** and FPKM value >1 **(G)** representation of DEGs related to necroptosis. See also Supplementary Figure S2.

To further investigate the epidermal pathways affected by HPV8 E7 expression, KEGG pathway enrichment analysis was conducted and revealed that HPV8 E7 overexpression mainly affected gene expression in the viral carcinogenesis pathway, transcriptional misregulation in cancer, and necroptosis pathways (Figure 3A). HPV8 E7 overexpression significantly inhibited SCIN expression but dramatically promoted the expression of 27 viral carcinogenesis-associated genes, including *JUN*, *H4C8*, *H2C21*, *CREB5,* and *H2BC8*, in NHEK cells (Supplementary Figure S2A; Figures 3B,C). On the other hand, in transcriptional misregulation in the cancer signaling pathway, only *MYCN* expression was notably decreased, and the expression of 20 genes, including *GADD45A*, *CXCL8*, *JMJD1C*, *RUNX1,* and *H3C14*, was significantly increased in NHEKs upon HPV8 E7 expression (Supplementary Figure S2B; Figures 3D,E). Further, HPV8 E7 overexpression significantly inhibited *TICAM2* expression but substantially promoted the expression of 15 genes, including *H2AC18*, *H2AC19*, *TNFAIP3*, *IL1A,* and *CYLD* in NHEK cells, all of which were crucial for the necroptosis signaling pathway (Supplementary Figure S2C; Figures 3F,G).

### HPV8 E7 Overexpression Significantly Affected Metabolism-Associated Gene Expression

**FIGURE 4:**
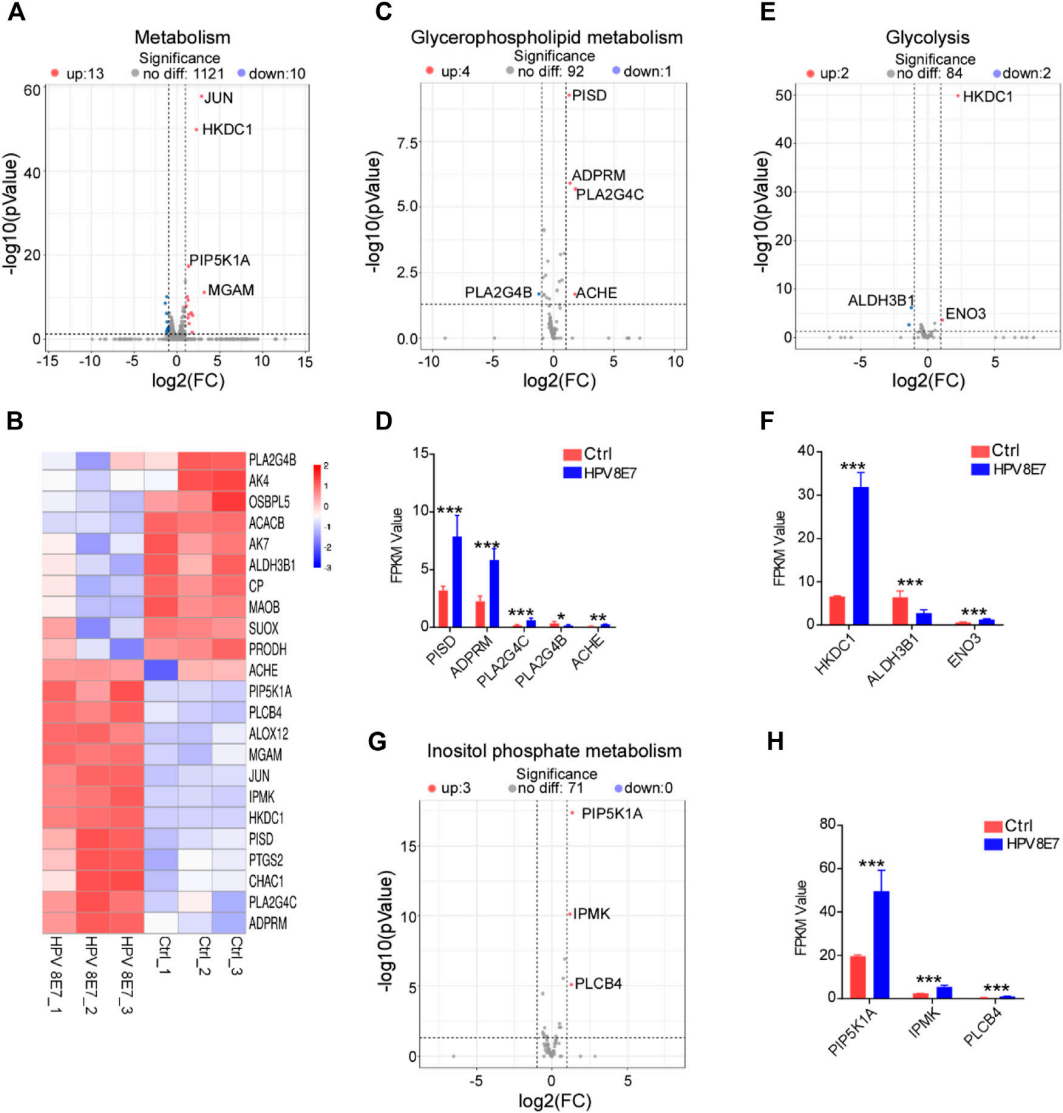
HPV8 E7 overexpression significantly affected metabolism-associated gene expression. **(A,B)** Volcano plot **(A)** and heatmap **(B)** representation of DEGs related to metabolism. **(C,D)** Volcano plot **(C)** and FPKM value > 1 **(D)** representation of DEGs related to glycerophospholipid metabolism. **(E,F)** Volcano plot **(E)** and FPKM value > 1 **(F)** representation of DEGs related to glycolysis. **(G,H)** Volcano plot **(G)** and FPKM value **(H)** representation of DEGs related to inositol phosphate metabolism. See also Supplementary Figure S3.

To evaluate the regulatory effects of HPV8 E7 on the metabolism status in epithelial cells, the cell metabolism-associated genes were enriched in NHEK cells after HPV8 E7 expression. As shown in Figures 4A,B, 23 DEGs associated with the metabolism pathway were detected in HPV8 E7-overexpressed NHEKs compared with controls, 11 of which were notably downregulated, such as *PLA2G4B*, *AK4*, *OSBPL5*, *ACACB*, and *AK7*; 12 genes were dramatically upregulated, including *JUN*, *HKDC1*, *PIP5K1A,* and *MGAM*. HPV8 E7 overexpression significantly inhibited *PLASG4B* expression but promoted *PISD*, *ADPRM*, *PLA2G4C*, and *ACHE* expression, which was associated with the glycerophospholipid metabolism pathway (Supplementary Figure S3A; Figures 4C,D). Furthermore, HPV8 E7 markedly decreased *ALDH3B1* expression, but increased *HKDC1* and *ENO3* expression, which was associated with the glycolysis metabolism pathway (Supplementary Figure S3B; Figures 4E,F). Moreover, HPV8 E7 overexpression significantly inhibited the expression of *PIP5K1A*, *IPMK,* and *PLCB4*, which was associated with the inositol phosphate metabolism pathway (Supplementary Figure S3C; Figures 4G,H). These results indicated that HPV8 E7 expression significantly altered the metabolism status in epidermal keratinocytes.

### HPV8 E7 Overexpression Significantly Affected Epigenetic-Associated Gene Expression

**FIGURE 5:**
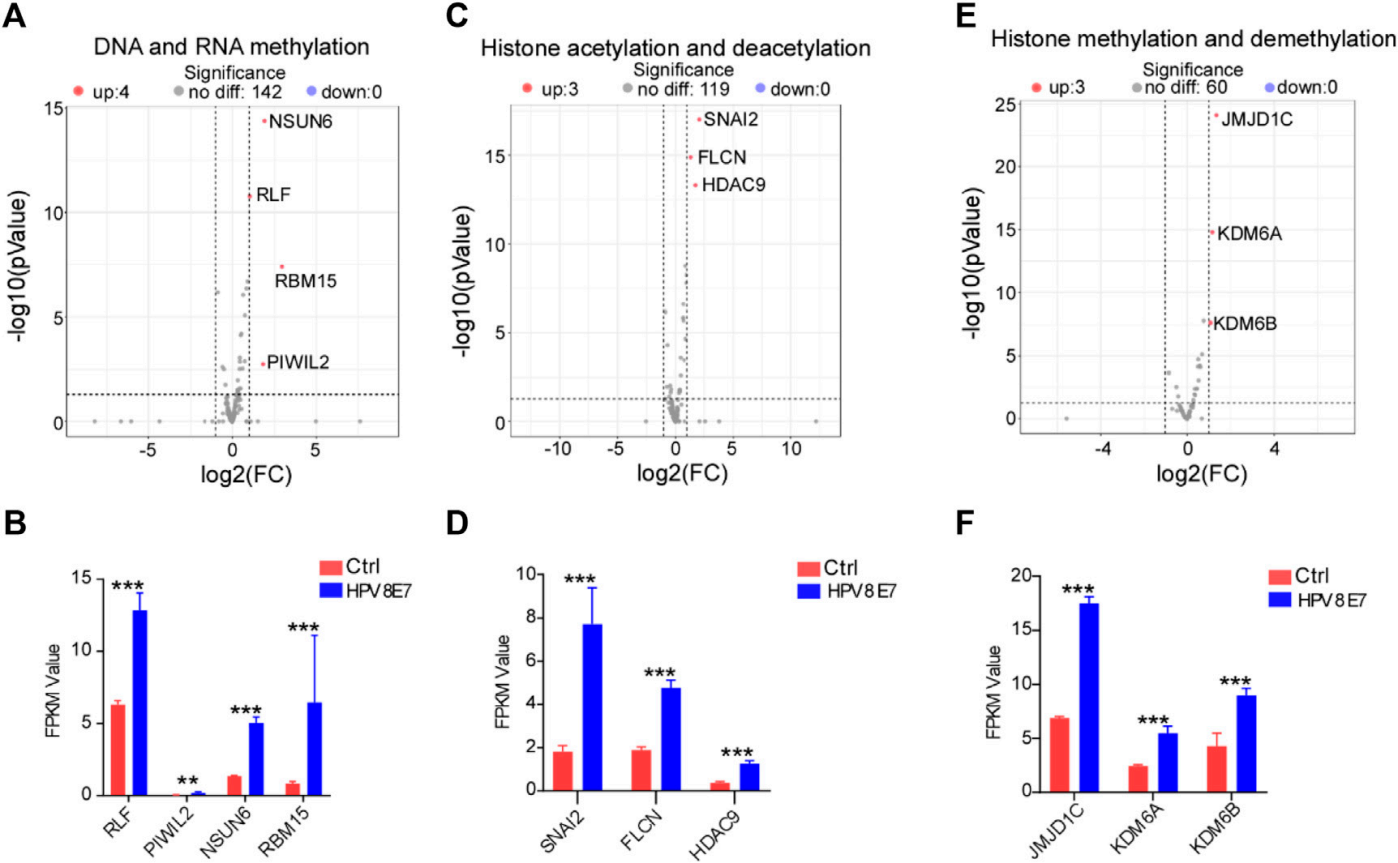
HPV8 E7 overexpression significantly affected epigenetic-associated gene expression. **(A,B)** Volcano plot **(A)** and FPKM value **(B)** representation of DEGs related to DNA and RNA methylation. **(C,D)** Volcano plot **(C)** and FPKM value **(D)** representation of DEGs related to histone acetylation and deacetylation. **(E,F)** Volcano plot **(E)** and FPKM value **(F)** representation of DEGs related to histone methylation and demethylation. See also Supplementary Figure S4.

To evaluate the regulatory effects of HPV8 E7 on epigenetic modifications in epithelial cells, cell epigenetic-associated genes were enriched in NHEK cells after HPV8 E7 expression. As shown in Supplementary Figure S4A; Figures 5A,B, DNA and RNA methylation-associated genes, such as *NSUN6*, *RLF*, *RBM15,* and *PIWIL2* were dramatically upregulated after HPV8 E7 expression. Furthermore, HPV8 E7 overexpression significantly promoted the expression of histone acetylation and deacetylation-associated genes, such as *SNAI2*, *FLCN,* and *HDAC9* (Supplementary Figure S4B; Figures 5C,D). Moreover, HPV8 E7 expression also notably increased the expression of the histone methylation and demethylation-associated genes, including *JMJD1C*, *KDM6A,* and *KDM6B* (Supplementary Figure S4C; Figures 5E,F). These results indicated that HPV8 E7 expression significantly increased the overall epigenetic modifications of epithelial cells.

### HPV8 E7 Overexpression Significantly Affected Immune Response-Associated Gene Expression

**FIGURE 6:**
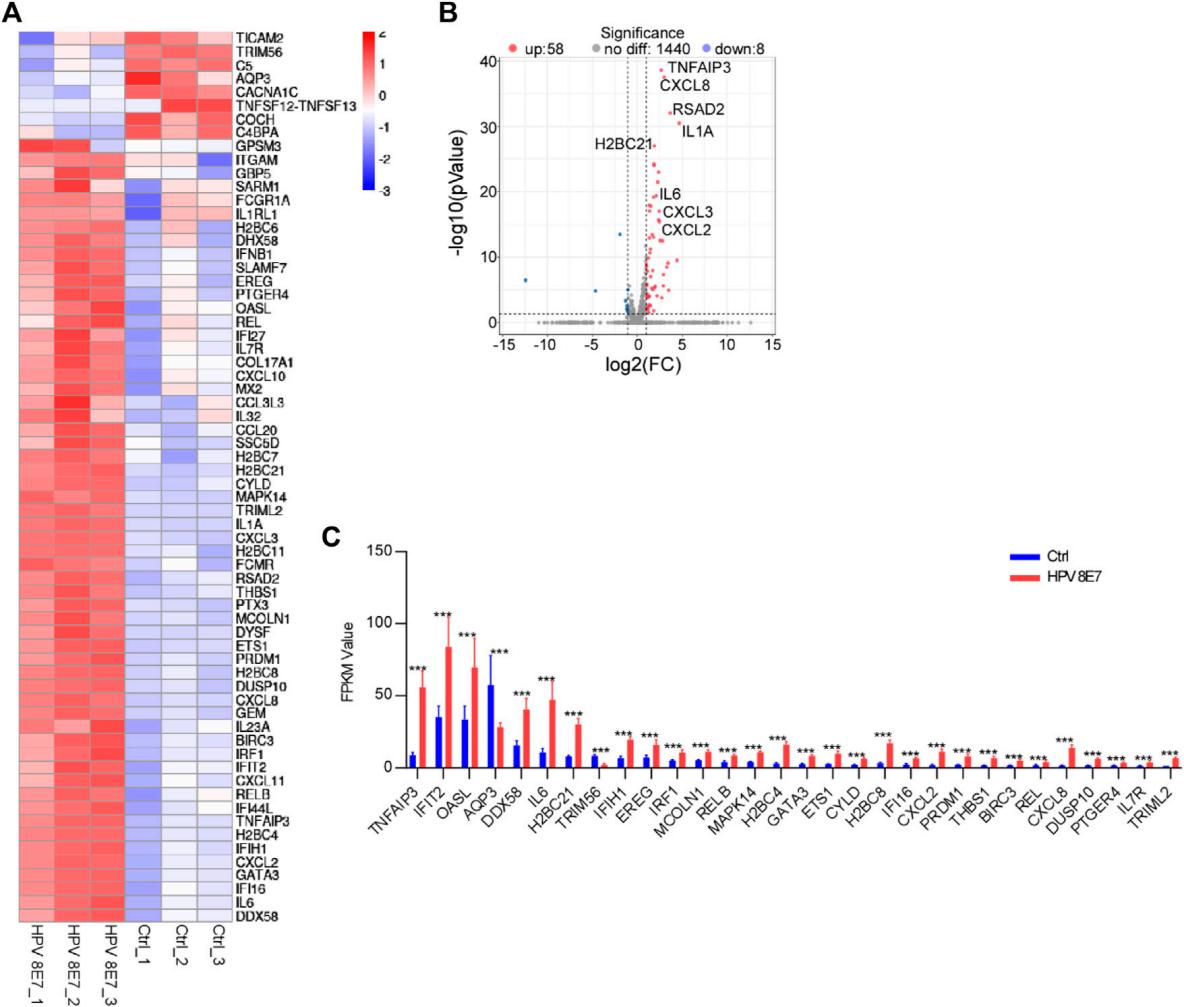
HPV8 E7 overexpression significantly affected immune response-associated gene expression. **(A)** Heatmap representation of the DEGs related to immunoregulation. **(B)** Volcano plot representation of the DEGs related to immunoregulation. **(C)** FPKM value (>1) of the DEGs related to immunoregulation.

Inflammatory response plays a pivotal role in the defense against viral infection. To evaluate the immune status after HPV8 E7 expression, immune response signaling pathway-associated genes were enriched. As shown in Figure 6A, 66 DEGs were detected in the NHEK cells after HPV8 E7 expression, among which, the expression of 8 genes, such as *TICAM2*, *TRIM56*, *C5*, *AQP3,* and *CACNA1C*, was significantly inhibited, but that of 58 genes, including *TNFAIP3*, *CXCL8*, *RSAD2*, *IL1A,* and *H2BC21* was substantially increased (Figures 6B,C). These results indicated that HPV8 E7 expression may affect an intense immune response in epithelial keratinocytes.

## Discussion

HPV infection, especially with high-risk subtypes, is associated with SCCs that arise in several different organs but share many common biological and immunological properties [18]. In cervical and anal cancers, HPV is the etiology for nearly 90% of malignancies, whereas only 30% of oropharyngeal, vaginal, vulvar, and penile cancers are driven by carcinogen exposure or other causes [19]. Mechanistically, HPV oncogenes not only directly inhibit the tumor suppressors p53 and Rb, but also suppress apoptosis and telomere shortening and promote genomic instability, angiogenesis, invasion, and metastasis to facilitate malignant transformation [20–22]. These properties render HPV infection, induced by factors other than sunlight exposure/UV light, or chemical irritants, an ideal model for the study of the potential mechanisms of HPV-associated cancers.

The functional evaluation of HPVs is far from complete, but currently, the two viral oncoproteins E6 and E7 have been the focus [23]. The HPV8 E6 oncoprotein mainly impairs the DNA damage repair pathway to facilitate carcinogenesis [24]. Moreover, carcinogenic effects are observed in epidermis HPV8 E7 oncoprotein-overexpressing transgenic mice [6]. *In vitro*, HPV8 E6 counters the cellular response to DNA damage and mitotic errors by destabilizing the functions of P300, a histone acetyltransferase [25]. Furthermore, a recent study finds that HPV8 E7-expressing human primary keratinocytes increase GADD34 and GDF15 expression to facilitate skin tumorigenesis by activating the Src kinase family members Fyn and Lyn [26]. Unfortunately, although the HPV8 E7 oncogene has been identified in more than 90% of SCCs and has been implicated in NMSCs, its exact role in human skin carcinogenesis has not been fully elucidated [3, 27, 28]. In this study, we took advantage of RNA-sequencing technology and the enrichment analysis of GO and KEGG pathways and found that 831 genes were significantly altered upon HPV8 E7 overexpression. Most of the DEGs could be clustered into nucleosomes, nucleosome assembly, viral carcinogenesis pathway, transcriptional misregulation in cancer, and necroptosis pathways. These are the first *in vitro* mechanistic data on the tumorigenicity of HPV8 E7 and may shed new light on the tumorigenic effects of HPV8 E7 expression.

The GO resource (http://geneontology.org) is a structured, computable database on gene function [29]. In the present study, most HPV8 E7 overexpression-induced genes could be clustered into protein heterodimerization activity and DNA binding (molecular function), nucleosomes (cellular component), and nucleosome assembly (biological process). Our results support the hypothesis that viral proteins interact and manipulate not only cytoplasmic components but also nuclear factors for their survival and propagation [30, 31]. The KEGG pathway database is a reference knowledge base that helps reconstruct molecular network systems [32]. In this study, the KEGG pathway enrichment analysis revealed that HPV8 E7 overexpression mainly affected gene expression in the viral carcinogenesis pathway, transcriptional misregulation in cancer, and necroptosis pathways, which were either important for HPV infection-driven carcinogenesis or could be an important marker for diagnosis and prognosis in HPV-related cancer [33, 34].

Metabolic reprogramming is an important hallmark in cancer [13]. However, the mechanisms of how HPV viral oncoproteins alter cell metabolism to satisfy their own energy demands and how this may contribute to tumorigenesis remain unknown [35]. Interestingly, the spare mitochondrial respiratory capacity of HPV8 E7-expressing cells is markedly increased, and this effect may be due to the direct interaction between HPV8 E7 and the mitochondrial ATP-synthase, which would dramatically result in decreased glycolytic activity [35]. Furthermore, the function of cell metabolism biomolecules, such as glucose, amino acids, and lipids, has been altered to promote cell proliferation, inhibit cell death, and affect genomic instability after HPV infection [9]. Indeed, in the current study, we not only detected DEG profiles in glycolysis pathways in HPV8 E7-expressing NHEKs, but also clustered DEGs into glycerophospholipid metabolism and inositol phosphate metabolism signaling pathways. Glycerophospholipids, the major components of cell membranes, play an important role in viral infection and are crucial for the morphogenesis of progeny virus and viral pathogenicity [36, 37]. Moreover, the G/G genotype and G allele of the inositol 1,4,5-trisphosphate 3-kinase C rs28493229 polymorphism may raise the risk of development of cervical SCC, and could be a potential marker for genetic susceptibility to CSCC [38].

It has been reported viral proteins can regulate epigenetic modifications to affect biological functions, such as cell proliferation and apoptosis [39]. Epigenetic mechanisms related to HPV infection include methylation changes to host and viral DNA and chromatin modification in host species [40]. In this study, we detected 10 epigenetic-associated genes significantly upregulated upon HPV8 E7 overexpression in epidermal keratinocytes, and these DEGs were clustered into DNA and RNA methylation, histone acetylation and deacetylation, and histone methylation and demethylation. HPV E7 promotes the expression of SUV39H1, which is a chromatin repressor and can inhibit the mRNA levels of RIG-I, cGAS, and STING genes by epigenetic silencing, and thereby blocks the interferon secretion in HPV-transformed cells [41]. Furthermore, changed methylated CpGs and regions reportedly regulate gene expression and affect prognosis in HPV-positive head and neck SCC [42]. Further, the epigenetic regulators histone deacetylases (1 and 2) are overexpressed in oncoprotein E6/E7-expressing cells, and histone deacetylase inhibitors could be a therapeutic intervention for skin cancer induced by HPV [43, 44]. An epigenome-wide analysis of HPV-driven common warts reveals that H3F3A, CDKN1A, and MAPK13 are the most differentially methylated genes [45]. Furthermore, HPV viral proteins can directly target histone methyltransferases for activity inhibition and modulate specific gene expression [39].

While the association between global immune compromise and HPV-related malignancies is well established, our understanding of how HPV perpetuates a state of immune suppression in the microenvironment remains limited [18]. Viral oncoproteins can control various cellular circuit programs, such as innate immunity to promote the proliferation of HPV-transformed cells [41]. However, the mechanisms of how persistent HPV8 infection affects the immune response to promote the carcinogenic process remain unknown. HPV E7 promotes the expression of H3K9-specific methyltransferase, which can regulate the host innate immune response to facilitate immune evasion [32]. However, in this study, we found many acute immune response genes, such as *TNFAIP3*, and those encoding interleukins and chemokines expression in HPV8 E7-expressing NHEKs, which were associated with the clearance of HPV infection.

However, the present study only provided the transcriptional regulation landscape of HPV8 E7-expressing NHEKs; our study limitation is that data interpretation is based only on an *in vitro* experiment and that it provides only theoretical evidence. Further, these experiments were conducted on a single keratinocyte, and whether the same results can be obtained from normal keratinocytes of other species remains uncertain; therefore, the exact tumorigenic mechanisms of HPV8 E7 need to be further corroborated.

## Conclusion

In conclusion, our results demonstrated the pro-carcinogenic effect of HPV8 expression in epithelial cells which could contribute to the regulatory effect of oncogene E7 on gene expression associated with nucleosomes, nucleosome assembly, viral carcinogenesis pathway, transcriptional misregulation in cancer, necroptosis pathways, epigenetic modifications, immune response, and metabolic status. Although our data are based on an *in vitro* experiment, it provides more effective evidence that the development of squamous cell carcinoma can be caused by HPV.

## Data Availability

The datasets presented in this study can be found in online repositories. The names of the repository/repositories and accession number(s) can be found in the article/Supplementary Material.
