# Supplementary material for: Gene Expression Profile Analysis of Human Epidermal Keratinocytes Expressing Human Papillomavirus Type 8 E7
Source: Pathol Oncol Res. 2022 May 18;28:1610176. doi: 10.3389/pore.2022.1610176 (PMC9156622; doi:10.3389/pore.2022.1610176)
Supplement: Supplementary file 1 [file DataSheet1.docx]

**Supplementary Data**

**Figure S1. GO enrichment analysis of DEGs affected by HPV8E7 overexpression**


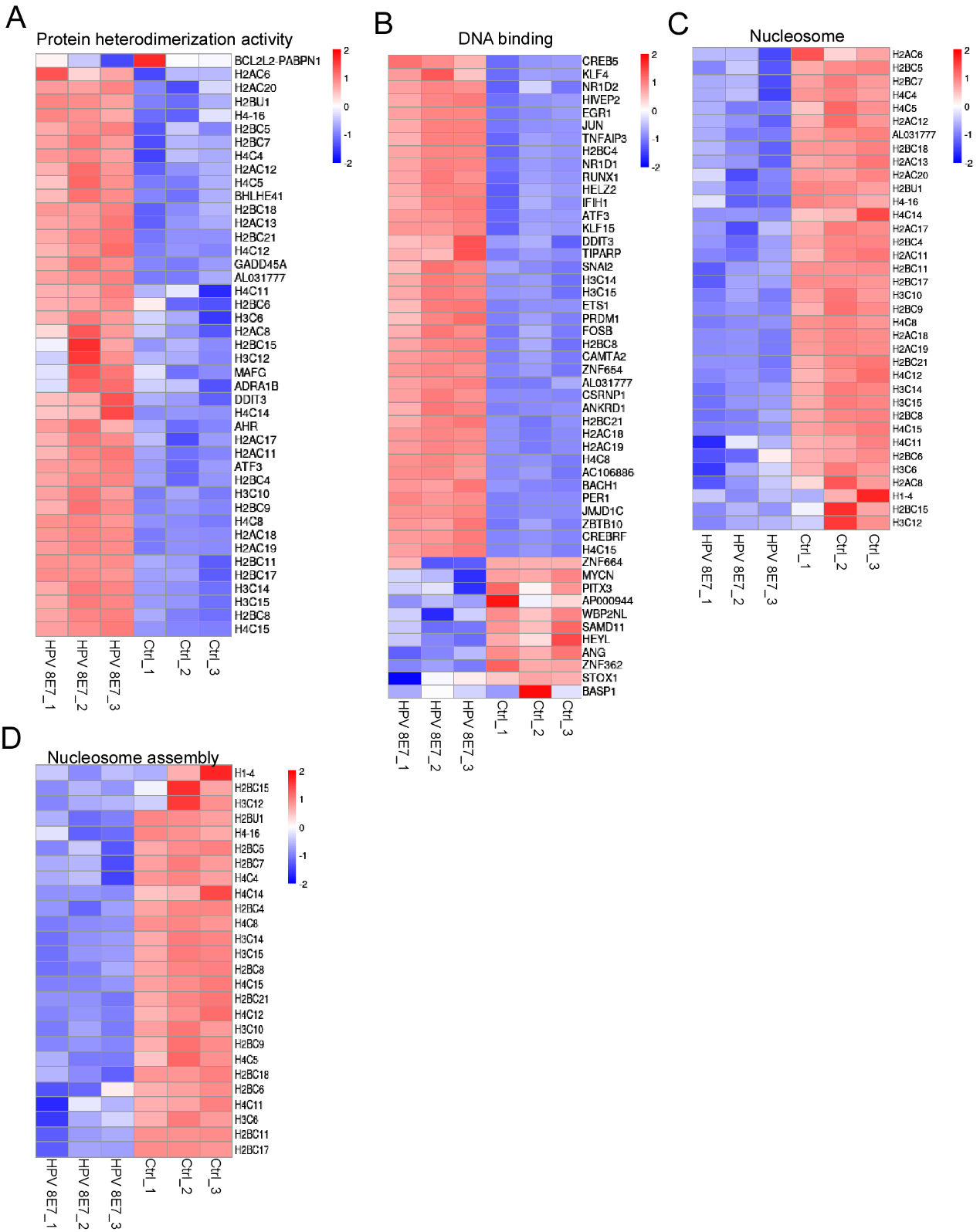


(**A**) Heatmap representation of the differentially expressed genes related to protein heterodimerization activity.

(**B**) Heatmap representation of the differentially expressed genes related to DNA binding.

(**C**) Heatmap representation of the differentially expressed genes related to nucleosome.

(**D**) Heatmap representation of the differentially expressed genes related to nucleosome assembly.

**Figure S2. KEGG pathway enrichment analysis of DEGs affected by HPV8E7 overexpression**

**
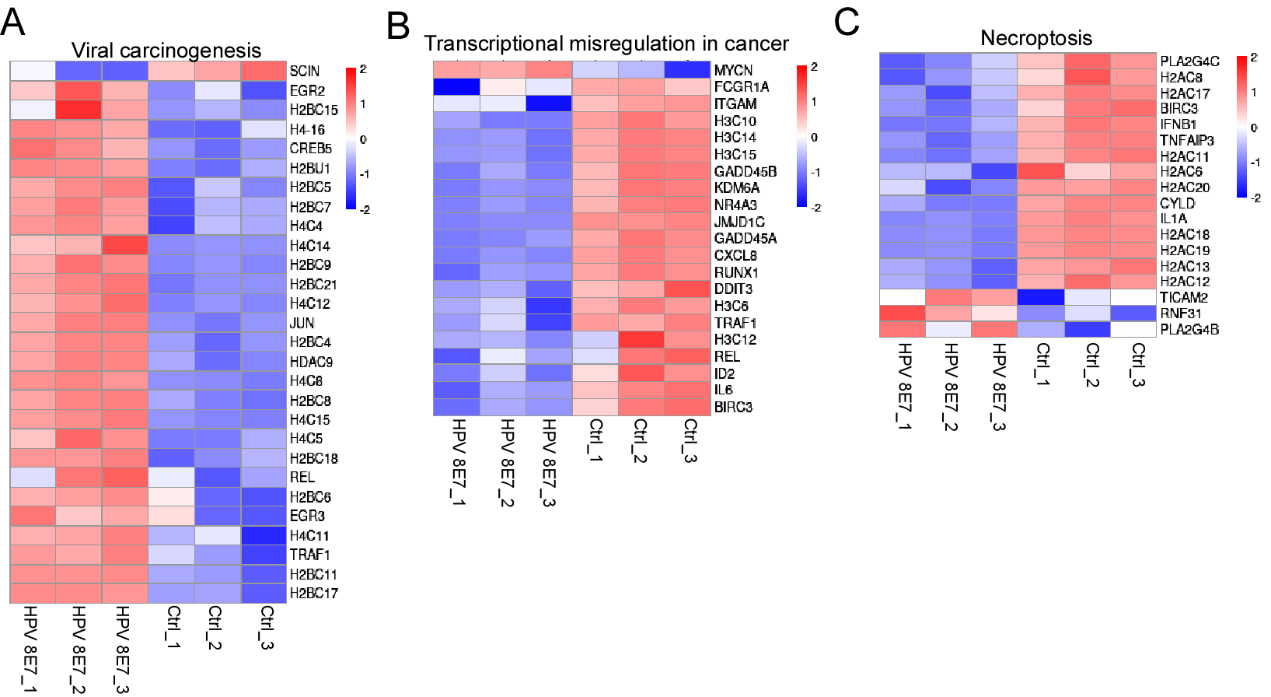
**

(**A**) Heatmap representation of the differentially expressed genes related to viral carcinogenesis.

(**B**) Heatmap representation of the differentially expressed genes related to transcriptional mis-regulation in cancer.

(**C**) Heatmap representation of the differentially expressed genes related to necroptosis.

**Figure S3. HPV8E7 overexpression significantly affects metabolism-associated genes expression**

**
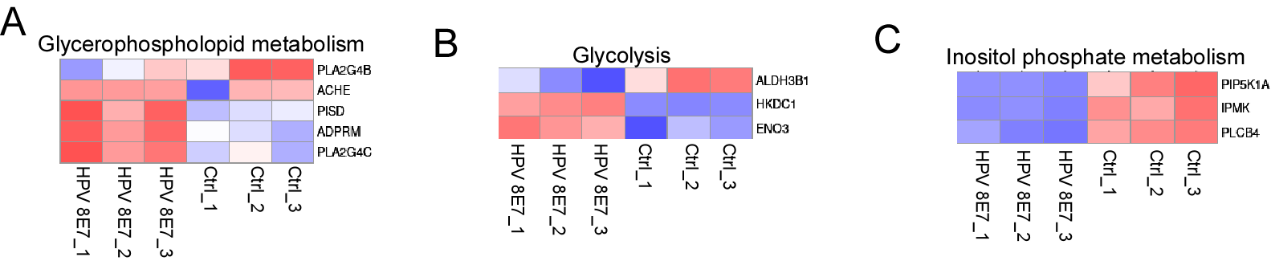
**

(**A**) Heatmap representation of the differentially expressed genes related to glycerophospholipid metabolism.

(**B**) Heatmap representation of the differentially expressed genes related to glycolysis.

(**C**) Heatmap representation of the differentially expressed genes related to inositol phosphate metabolism.

**Figure S4. HPV8E7 overexpression significantly affected epigenetics-associated genes expression**

**
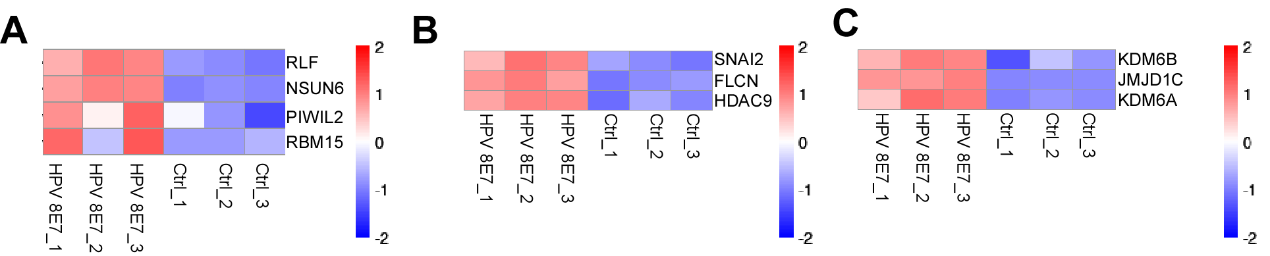
**

(**A**) Heatmap representation of the differentially expressed genes related to DNA and RNA methylation.

(**B**) Heatmap representation of the differentially expressed genes related to histone acetylation and deacetylation.

(**C**) Heatmap representation of the differentially expressed genes related to histone methylation and demethylation.
